# Standardized Patient Communication and Low-Value Spinal Imaging: A Randomized Clinical Trial

**DOI:** 10.1001/jamanetworkopen.2024.41826

**Published:** 2024-11-06

**Authors:** Joshua J. Fenton, Camille Cipri, Melissa Gosdin, Daniel J. Tancredi, Anthony Jerant, Carly Ann Robinson, Guibo Xing, Ilona Fridman, Gary Weinberg, Andrew Hudnut

**Affiliations:** 1 Department of Family and Community Medicine, University of California, Davis, Sacramento; 2 The Center for Healthcare Policy and Research, University of California, Davis, Sacramento; 3 School of Medicine, University of California, Davis, Sacramento; 4 Department of Pediatrics, University of California, Davis, Sacramento; 5 Lineberger Comprehensive Cancer Center, University of North Carolina, Chapel Hill; 6 Sutter Institute for Medical Research, Sacramento, California

## Abstract

**Importance:**

Acute back pain is a common reason for primary care visits and often results in low-value spinal imaging.

**Objective:**

To evaluate the effect of a standardized patient-delivered intervention on rates of low-value spinal imaging among primary care patients with acute low back pain.

**Design, Setting, and Participants:**

In this randomized clinical trial, physicians or advanced practice clinicians were recruited from March 22 to August 5, 2021, from 10 adult primary care or urgent care clinics in Sacramento, California. The intervention period was from May 1, 2021, to March 30, 2022, with follow-up from October 28, 2021, to June 30, 2023. Analyses were performed from April 1 to June 25, 2024.

**Intervention:**

Clinicians were randomized 1:1 to intervention or control. Intervention clinicians received 3 simulated office visits, each with a standardized patient instructor (SPI) portraying a patient with acute uncomplicated back pain. At each visit, SPIs provided clinician feedback guided by a 3-step model: (1) set the stage for deferred imaging by building trust, (2) convey empathy, and (3) communicate optimism while advocating watchful waiting without imaging. Control clinicians received no intervention.

**Main Outcomes and Measures:**

The primary outcome was lumbar spinal imaging completion within 90 days of acute low back pain visits, with study clinicians assessed up to 18 months of follow-up. Secondary outcomes were cervical spine imaging completion after acute neck pain visits, any imaging completion after an adult visit, patient experience ratings of clinicians (scale range, 0-100), and use of targeted communication skills during an audio-recorded standardized patient evaluation visit at median follow-up of 16.8 months (range, 14.1-18.0 months).

**Results:**

The analysis included 53 clinicians; mean (SD) age was 46.7 (1.0) years, and 35 (66.0%) reported female gender. A total of 25 were in the intervention group and 28 in the control group. After adjustment for prerandomization rates, patients with acute low back pain who saw intervention and control clinicians during follow-up had similar rates of lumbar imaging (194 of 1234 clinic visits [15.7%] vs 226 of 1306 clinic visits [17.3%]; adjusted ratio of postintervention vs preintervention odds ratios [AORR], 1.00; 95% CI, 0.72-1.40). Adjusted follow-up rates of imaging for acute neck pain (AORR, 1.16; 95% CI, 0.83-1.63) and overall imaging (AORR, 1.07; 95% CI, 0.97-1.19) were not significantly different among patients of intervention and control clinicians. Intervention and control clinicians had similar mean (SD) patient experience ratings during follow-up (88.6 [28.7] vs 88.8 [28.3]; adjusted mean difference-in-differences, −1.0; 95% CI, −3.0 to 0.9). During audio-recorded standardized patient visits, intervention clinicians had significantly better ratings than controls on eliciting the patient’s perspective (adjusted standardized difference [ASD], 0.62; 95% CI, 0.05-1.19) and conveying empathy (ASD, 1.16; 95% CI, 0.55-1.77).

**Conclusions and Relevance:**

In this randomized clinical trial of an educational intervention using simulated office visits to encourage a watchful waiting approach for acute low back pain, the intervention had no significant effect on low-value spinal imaging rates or patient experience ratings.

**Trial Registration:**

ClinicalTrials.gov Identifier: NCT04255199

## Key Points

**Question:**

Can a standardized patient (SP)–delivered communication intervention reduce low-value lumbar spinal imaging among patients with uncomplicated low back pain?

**Findings:**

In this randomized clinical trial involving 53 primary care clinicians in 10 clinics, intervention clinicians received 3 visits over a 6-month period from SP instructors emphasizing a watchful waiting approach for patients with uncomplicated low back pain. During an 18-month follow-up period, the intervention had no significant effect on the rate of low-value lumbar spinal imaging within 90 days of patient visits.

**Meaning:**

The findings suggest that educational interventions emphasizing clinician communication are unlikely to reduce rates of low-value spinal imaging among primary care clinicians.

## Teaser

This randomized clinical trial investigates whether a clinician communication intervention emphasizing a watchful waiting approach reduces the rate of low-value lumbar spinal imaging among primary care patients with acute low back pain.

## Introduction

Acute low back pain is a common reason for primary care visits. Patients with acute back pain often present with disability and distress. Although acute back pain has a favorable prognosis, patients frequently expect to receive diagnostic imaging, and primary care clinicians may feel pressure to obtain imaging to sustain patient trust and satisfaction.^1^ For patients with uncomplicated acute low back pain, spinal imaging typically yields no helpful diagnostic information yet poses risks of false-positive findings, patient labeling and anxiety,^2^ and unnecessary treatments.^3^ Early imaging for low back pain is often examined in studies of low-value care, which is care that augments costs but yields few or no health benefits.^4,5,6,7,8,9^ The National Committee for Quality Assurance (NCQA) has endorsed spinal imaging in the initial 28 days of an acute back pain episode as a measure of overuse in primary care.^10^

While the Choosing Wisely movement increased physician awareness of low-value care, it has been associated with small differences in the use of low-value imaging for acute low back pain.^4,11^ While an earlier US trial found that patient education may reassure patients with back pain that imaging could be safely omitted,^12^ clinician-directed interventions, including precommitment to avoiding low-value care, have had little or no effect on the use of low-value spinal imaging.^13,14^ More impactful approaches to reducing the use of low-value spinal imaging are needed.

Watchful waiting advice has been an effective strategy to reducing low-value treatments.^15^ In a Dutch randomized clinical trial, a watchful waiting strategy was acceptable to patients with unexplained symptoms and reduced diagnostic blood testing.^16^ In an observational study of primary care visits, primary care clinicians who advised watchful waiting when patients requested low-value testing were 40% less likely to order requested tests than those who did not.^17^

We developed an educational intervention delivered by standardized patient instructors (SPIs) that was designed to boost primary care clinician skill in delivering a watchful waiting message to patients with acute low back pain. SPI-based interventions have improved clinician communication regarding HIV risk, chronic disease self-management, smoking, informed consent, and advanced cancer care.^18,19,20,21,22^ In this randomized clinical trial, we tested the effectiveness of an SPI-delivered intervention for reducing rates of early imaging among patients with acute back pain. We also examined whether the intervention had effects on imaging for acute neck pain, overall diagnostic imaging, and patient experience.

## Methods

We conducted a randomized clinical trial with primary care clinicians randomized 1:1 to intervention and control groups. The trial protocol was preregistered at ClinicalTrials.gov (NCT04255199), is given in Supplement 1, and was previously published.^23^ The study was approved by the University of California, Davis, institutional review board. We obtained oral informed consent from all participants. This study adhered to the Consolidated Standards of Reporting Trials (CONSORT) reporting guideline.^24^

### Participants

From March 22 to August 5, 2021, we recruited primary care physicians or advanced practice clinicians in 2 integrated health systems in the Sacramento, California, region. Clinicians were eligible if they were in an adult primary care or urgent care practice (≥50% full-time equivalent), were practicing in the same system during the prior 18 months, and had no plans to leave the practice in the next 2 years. Clinicians completed baseline questionnaires assessing demographics, years in practice, and stress from uncertainty.^25^

### Randomization and Blinding

Clinicians were randomized using permuted block randomization stratified by system and primary vs urgent care. Allocation was concealed in sequentially labeled, sealed opaque envelopes, which the study coordinator opened when each clinician enrolled. As the coordinator (C.C.) was responsible for scheduling the standardized patient interventions, the coordinator and standardized patient staff were not blinded to allocation. We also could not blind study clinicians to intervention and control assignments.

### Experimental Conditions

Clinicians randomized to the control group received no intervention during the trial. From May 1, 2021, to March 30, 2022, clinicians randomized to the intervention group received three 20-minute, in-person office visits scheduled over a 6-month period during normal clinic hours, each with a trained SPI. Clinicians were aware that they were scheduled to see the SPI.

During intervention visits, SPIs spent 10 to 12 minutes portraying patients with acute uncomplicated back pain based on prespecified roles. During this time, SPIs assessed clinicians’ performance based on a 3-step intervention model for communicating a watchful waiting message regarding spinal imaging (eTable 1 in Supplement 2). The model was grounded in sociopsychological theory of motivation and message personalization,^26,27,28^ prior literature and preliminary studies,^12,22,29,30,31^ key informant interviews with clinicians, and patient focus groups. The 3 steps were to (1) set the stage for deferred imaging by building trust, (2) convey empathy, and (3) communicate optimism while advocating a plan without imaging. During the final 8 to 10 minutes of visits, SPIs provided formative feedback to clinicians based on their adherence to the model during the initial part of the visit. During visits, SPIs referred to a printed handout depicting the intervention model (eFigure in Supplement 2) that included examples for how clinicians might effectively communicate specific messages, and they left the handout with clinicians at the end of the visits. At each visit, SPIs briefly referred to red flags that would constitute indications for imaging.

The 3-visit dose of the intervention was similar to prior SPI interventions that successfully improved clinician communication.^21,22^ As intervention visits occurred during the COVID-19 pandemic, SPIs wore face coverings during visits and the physical examination was omitted by providing examination findings on a printed card. Patient data, such as medical history and vital signs, were also provided on printouts. During the Delta wave of the pandemic (late 2021), SPI visits were temporarily paused, resulting in a delayed second or third visit for some clinicians. The SPIs audio-recorded all visits so that standardized patient trainer (G.W.) could monitor intervention fidelity using a checklist.

### Outcomes

We used electronic medical record data to ascertain primary and secondary imaging outcomes. The primary outcome was completion of lumbar spinal imaging (radiography, magnetic resonance imaging [MRI], or computed tomography [CT]) within 90 days of visits with study clinicians by adult patients with uncomplicated acute back pain during an 18-month follow-up period (from October 28, 2021, to June 30, 2023) after the final intervention visit. For control clinicians, we assigned a postintervention date that was randomly selected and distributed similarly to the final intervention dates for intervention clinicians. Uncomplicated acute back pain visits were identified using criteria similar to those used for the NCQA low-value spinal imaging measure.^10^ Detailed methods for specifying the electronic medical record–derived imaging outcomes are given in the eMethods and eTables 2 to 4 in Supplement 2.

Secondary imaging outcomes included (1) an analogous measure of the completion of cervical spinal imaging among adult patients with acute neck pain, (2) completion of lumbar or cervical imaging with either MRI or CT, and (3) completion of any diagnostic imaging among adult patients who had visits with study clinicians. We ascertained preintervention imaging outcomes among patients seen by study clinicians during a 24-month prerandomization period to enable adjustment for baseline imaging propensity.

As a secondary outcome, we obtained patient experience data for adult respondents after visits with study clinicians during the study period. Each health system routinely surveys patients using standardized questions about recent visit experience. For responses linked to study visits, we extracted data on patients’ clinician ratings and combined item ratings into a summary scale ranging from 0 (worst) to 100 (best). Detailed methods for summarizing the patient experience data are provided in the eMethods in Supplement 2.

Approximately 9 months after randomization, intervention and control physicians received a scheduled audio-recorded visit with an SPI portraying a patient with acute low back pain. Clinicians received no training during these visits. As a secondary outcome, we assessed clinician use of targeted communication techniques during this visit. Guided by a coding manual, trained and blinded coders (including C.C., C.A.R., and G.W.) independently coded the transcripts using an adaptation of the validated Four Habits Model,^32^ which mapped closely to the 3 intervention steps. Interrater reliability for coding within each of the Four Habits Model domains was good to excellent (weighted κ, 0.60-0.71). Six months after the final SPI visits, we surveyed study clinicians regarding the use of watchful waiting with patients with back or neck pain and, for intervention clinicians, the quality, acceptability, and utility of the SPI training.

### Sample Size

Targeted sample size was based on a power analysis assessing a 2-tailed test of significance for a postintervention absolute difference of 7% in the primary lumbar imaging outcome (adjusted for baseline rates), assuming 25% incidence in the control group; within-clinic and within-clinician correlations of 1% and 4%, respectively; and α = 5%. With 8 clinics and 6 clinicians per clinic (48 clinicians) and 92 patients with acute low back pain per clinician (57 preintervention and 35 postintervention), the study would have 80.1% power. To retain the targeted sample size of 48 clinicians, we sought to enroll 55 clinicians.

### Statistical Analysis

Analyses were conducted from April 1 to June 25, 2024, using the intention-to-treat principle. For imaging and patient experience outcomes, units of analysis were patient visits, with visits classified as preintervention and postintervention and with the preperiod encompassing the 24 months prior to randomization and the postperiod, the 18 months after the final intervention visit or until the end of data collection on June 1, 2023. Outcomes were modeled using generalized linear mixed models with random effects for clinicians and adjustment for preintervention period vs postintervention period, patient age and sex, site, and whether visits were in-person or telemedicine. We also adjusted for clinician age, sex, and stress from uncertainty^25^ because these were unbalanced after randomization. Generalized linear mixed models used a logistic link and binary distribution for binary imaging outcomes and an identity link and gaussian distribution for the continuous patient experience outcome. We evaluated intervention effects by testing for the significance of an interaction term between a categorical variable for intervention group and preintervention vs postintervention period. Using fitted models, we quantified intervention effects by estimating the adjusted ratio of postintervention and preintervention odds ratios (AORR) for binary outcomes or adjusted mean difference-in-differences (AMDD) based on the regression coefficients for the interaction term contrasting intervention vs control group differences in postintervention vs preintervention differences. For lumbar imaging, we planned a subgroup analysis for patients aged 18 to 65 years, consistent with the NCQA overuse measure.^10^ We performed a post hoc analysis to model intervention effects on patient experience scores within the subgroup of patient experience respondents who had visits with study clinicians for acute low back pain.

For coded data on clinician use of targeted communication behaviors during audio-recorded standardized patient visits, we created standardized summary scales by averaging scores across items within each domain of the Four Habits Model (Cronbach α range, 0.28-0.84). We then used linear regression to estimate mean between-group differences in outcomes, with these differences expressed as adjusted standardized differences (ASDs), by dividing each by the residual mean square error for the outcome.

Hypothesis tests were 2-sided, with α = .05 and effect sizes reported with 95% CIs. Analyses were conducted with either SAS, version 9.4 (SAS Institute Inc), or Stata MP, version 15.1 (StataCorp LLC).

## Results

Of 57 clinicians within 10 clinics, 27 were randomized to the intervention and 30 to control. Among the 53 clinicians included in final analyses, the mean (SD) age was 46.7 (1.0) years; 35 (66.0%) reported female gender, and 18 (34.0%) were male. A total of 49 (92.5%) were primary care physicians, 2 (3.8%) were physician assistants or nurse practitioners in primary care, and 2 (3.8%) were urgent care clinicians practicing at a primary care site. Compared with control clinicians, clinicians randomized to the intervention group were older, less likely to be female, and experienced less stress from uncertainty (Table 1). Of 25 clinicians randomized to the intervention, all received 3 intervention SPI visits. Of the 53 clinicians, 50 (94.3%) completed an audio-recorded standardized patient visit at approximately 9 months of follow-up. The median postintervention follow-up was 16.8 months (range, 14.1-18.0 months) (Figure). During the study period, clinicians had a mean (SD) of 101 (47) acute back pain visits, with a mean (SD) of 53.1 (24.3) visits in the 24-month prerandomization period and 48.3 (30.8) visits during follow-up.

**Table 1.** Characteristics of Randomized Clinicians

Characteristic	Clinicians (N = 53)^a^	
	Intervention (n = 25)	Control (n = 28)
Age, mean (SD), y	49.2 (6.9)	44.4 (7.6)
Gender		
Female	13 (52.0)	22 (78.6)
Male	12 (48.0)	6 (21.4)
Time in practice, mean (SD), y	19.8 (7.8)	14.0 (8.0)
Full-time	10 (40.0)	11 (39.3)
Tolerance of uncertainty, mean (SD)^b^		
Stress from uncertainty	40.4 (13.2)	46.4 (9.3)
Reluctance to disclose uncertainty	21.6 (6.1)	21.8 (7.0)

^a^ Data are presented as number (percentage) of clinicians unless otherwise indicated.

^b^ Tolerance of uncertainty scale as described by Gerrity et al^25^; scores range from 13 to 78 for stress from uncertainty and 9 to 39 for reluctance to disclose uncertainty, with higher scores indicating greater stress or reluctance, respectively.

**Figure.**
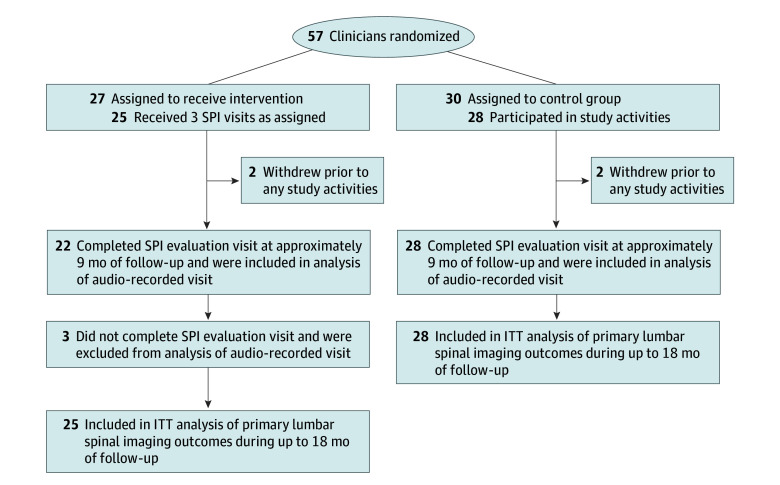
CONSORT Flow Diagram of Clinicians in Intervention and Control Groups ITT indicates intention to treat; SPI, standardized patient instructor.

During the postrandomization period, clinicians in the intervention and control groups had similar rates of lumbar imaging completion within 90 days of acute back pain visits (194 of 1234 [15.7%] vs 226 of 1306 [17.3%]), with no significant difference in the adjusted intervention vs control ratio of postrandomization vs prerandomization ORs (AORR, 1.00; 95% CI, 0.72-1.40) (Table 2). The intervention was also not associated with a significant difference in lumbar imaging completion in the planned subgroup analysis of patients aged 18 to 65 years (AORR, 1.25; 95% CI, 0.86-1.81).

**Table 2.** Primary and Secondary Imaging Outcomes

Presentation, outcome	Encounters, No. (%)				AOR (95% CI)^a^^,^^b^		AORR (95% CI)^a^^,^^c^	*P* value
	Preintervention		Postintervention					
	Intervention	Control	Intervention	Control	Intervention	Control		
**Low back pain**								
Encounters, No.	1355	1461	1234	1306	NA	NA	NA	NA
Primary outcome: lumbar imaging completed within 90 d	243 (17.9)	294 (20.1)	194 (15.7)	226 (17.3)	1.01 (0.77-1.32)	1.00 (0.83-1.22)	1.00 (0.72-1.40)	.99
Secondary outcomes								
Lumbar imaging ordered during encounter	342 (25.2)	390 (26.7)	262 (21.2)	298 (22.8)	0.93 (0.70-1.23)	0.95 (0.76-1.18)	0.98 (0.68-1.40)	.91
Lumbar MRI or CT ordered	156 (11.5)	163 (11.2)	119 (9.6)	151 (11.6)	0.94 (0.60-1.46)	1.19 (0.90-1.56)	0.79 (0.47-1.33)	.38
Lumbar MRI or CT completed within 90 d	109 (8.0)	111 (7.6)	76 (6.2)	98 (7.5)	0.79 (0.58-1.08)	1.04 (0.78-1.39)	0.76 (0.50-1.16)	.20
**Neck pain**								
Encounters, No.	1285	1289	1164	1180	NA	NA	NA	NA
Secondary outcomes								
Cervical imaging ordered	367 (28.6)	419 (32.5)	340 (29.2)	357 (22.8)	1.10 (0.85-1.43)	0.99 (0.84-1.17)	1.11 (0.81-1.51)	.66
Cervical imaging completed within 90 d	259 (20.2)	315 (24.4)	245 (21.0)	259 (21.9)	1.11 (0.85-1.44)	0.96 (0.76-1.20)	1.16 (0.83-1.63)	.87
Cervical MRI or CT ordered	114 (8.9)	127 (9.9)	131 (11.3)	104 (8.8)	1.33 (0.97-1.82)	0.87 (0.67-1.14)	1.52 (1.02-2.28)	.04
Cervical MRI or CT completed within 90 d	73 (5.7)	84 (6.5)	91 (7.8)	63 (5.3)	1.32 (0.95-1.83)	0.76 (0.53-1.07)	1.75 (1.09-2.81)	.02
**All adult primary care**								
Encounters, No.	116 327	123 770	86 045	98 954	NA	NA	NA	NA
Secondary outcomes								
Any imaging ordered	20 558 (17.7)	21 276 (17.2)	17 048 (19.8)	17 798 (18.0)	1.15 (1.08-1.22)	1.11 (1.02-1.20)	1.04 (0.94-1.15)	.42
Any imaging completed within 90 d	14 767 (12.7)	16 345 (13.2)	12 216 (14.2)	13 444 (13.6)	1.15 (1.08-1.22)	1.07 (0.98-1.17)	1.07 (0.97-1.19)	.19

Abbreviations: AOR, adjusted odds ratio; AORR, adjusted ratio of postintervention vs preintervention odds ratios; CT, computed tomography; MRI, magnetic resonance imaging; NA, not applicable.

^a^ Adjusted for study site, patient age, patient sex, in-person vs telephone or video visit, and physician baseline characteristics (age, sex, years in practice, and tolerance of uncertainty). Encounters were nested within 25 intervention and 28 control clinicians.

^b^ Postrandomization vs prerandomization.

^c^ Intervention vs control ratio of postrandomization vs prerandomization odds ratios.

The intervention was not associated with significant postrandomization reductions in secondary imaging outcomes, including completion of cervical imaging after acute neck pain visits (AORR, 1.16; 95% CI, 0.83-1.63), completion of lumbar or cervical imaging with MRI or CT, or completion of any imaging after adult visits (AORR, 1.07; 95% CI, 0.97-1.19) (Table 2). Compared with control, the intervention was associated with a significant increase in the rate of cervical MRI or CT completion after acute neck pain visits (AORR, 1.75; 95% CI, 1.09-2.81).

During the prerandomization and postrandomization periods, patient experience scale scores were similar among intervention and control clinicians. During the prerandomization period, the mean (SD) score was 89.3 (27.3) for 3433 encounters in the intervention group and 88.3 (28.0) for 3419 encounters in the control group (adjusted mean difference [AMD], −0.6; 95% CI, −1.8 to 0.6). During the postrandomization period, the mean (SD) score was 88.6 (28.7) for 7430 encounters in the intervention group and 88.8 (28.3) for 7484 encounters in the control group (AMD, 0.4; 95% CI, −1.1 to 1.9) (AMDD, −1.0; 95% CI, −3.0 to 0.9; *P* = .30). In a post hoc analysis of patient experience after acute back pain visits with study clinicians (n = 267), mean patient experience scores also did not differ significantly among intervention and control clinicians (mean [SD] postintervention score: 91.9 [25.4] vs 84.0 [34.3]; AMDD, 1.9; 95% CI, −18.2 to 21.9).

During audio-recorded standardized patient visits at approximately 9 months of follow-up, intervention clinicians had significantly higher ratings compared with controls on 2 of the 4 habits in the Four Habits Model (Table 3). For eliciting the patient perspective (habit 2), the ASD in scale score was 0.62 (95% CI, 0.05-1.19), and for conveying empathy (habit 3), the ASD was 1.16 (95% CI, 0.55-1.77). There were no significant differences in investing in the beginning (habit 1) or investing in the end (habit 4). Notably, clinicians in the intervention and control groups recommended a conservative approach and conveyed a watchful waiting message at most of the SPI visits.

**Table 3.** Ratings of Clinician Communication Behaviors During Office Visits With Standardized Patients With Back Pain

Item within habits domain	Score, mean (SD)^a^		ASD (95% CI)^c^	*P* value
	Intervention (n = 22)^b^	Control (n = 28)		
Habit 1, invest in beginning (Cronbach α = .28)				
Overall	7.1 (1.6)	6.7 (1.4)	0.25 (−0.26 to 0.75)	.33
Expansion of concerns	4.7 (0.9)	4.9 (0.5)	NA	NA
Eliciting full agenda	2.4 (1.1)	1.9 (1.1)	NA	NA
Habit 2, elicit patient perspective (Cronbach α = .53)				
Overall	10.2 (2.7)	9.3 (2.9)	0.62 (0.05 to 1.19)	.04
Eliciting patient’s understanding	3.7 (1.2)	3.4 (0.8)	NA	NA
Eliciting patient’s goals	3.3 (1.1)	2.9 (1.3)	NA	NA
Attention to psychosocial issues	3.2 (1.5)	2.9 (1.8)	NA	NA
Habit 3, convey empathy (Cronbach α = .84)				
Overall	10.5 (3.3)	6.9 (3.5)	1.16 (0.55 to 1.77)	<.001
Expression of emotion	3.5 (1.5)	2.4 (1.3)	NA	NA
Acceptance of feelings	3.9 (1.5)	2.4 (1.5)	NA	NA
Identification of feelings	3.2 (1.2)	2.0 (1.2)	NA	NA
Habit 4, invest in the end (Cronbach α = .40)				
Overall	22.1 (2.6)	22.1 (2.5)	0.10 (−0.50 to 0.70)	.74
Using patient’s concerns as frame of reference	4.1 (1.2)	3.6 (1.6)	NA	NA
Convey optimism	4.5 (0.9)	4.4 (0.9)	NA	NA
Advocate conservative treatment plan	4.9 (0.4)	4.9 (0.4)	NA	NA
Watchful waiting message	4.4 (1.0)	4.4 (0.9)	NA	NA
Convey plans for follow-up	4.2 (1.2)	4.7 (0.7)	NA	NA

Abbreviations: ASD, adjusted standardized difference; NA, not applicable.

^a^ Item scores range from 1 to 5, with higher scores indicating clinician communication that is more aligned with targeted behavior.

^b^ Three of the 25 clinicians randomized to the intervention did not complete the standardized patient evaluation visit at approximately 9 months of follow-up, so were not included in the analysis.

^c^ Computed by dividing the regression coefficient for the standardized scale score for each domain by the root mean square error of the dependent variable in the regression models. Models were adjusted for physician age, sex, and stress from uncertainty.^25^

In a posttrial survey, intervention clinicians rated the overall quality of the SPI training highly. Compared with control clinicians, they reported significantly greater confidence and frequency of using a watchful waiting approach for back pain (eTables 5 and 6 in Supplement 2).

## Discussion

In this randomized clinical trial, we found that an SPI-based intervention did not yield significant changes in the primary outcome of low-value spinal imaging among patients with acute low back pain seen by study clinicians. We also found no beneficial effects of the intervention on secondary imaging outcomes among patients with acute neck pain or on imaging rates or patient experience among the overall population of adult patients seen by study clinicians during the postintervention period.

SPI interventions are appealing because they can be embedded within clinical workdays and, as in this study, are often rated favorably by clinicians.^21,31^ Our primary care–based SPI intervention comprised three 20-minute office visits during which SPIs presented the intervention content and provided personalized feedback to clinicians. A slightly longer SPI intervention targeting oncologists improved patient-centered communication among patients with advanced cancer but had no effect on health care utilization.^22^ It is possible that the current study’s intervention was too limited in intensity to achieve meaningful changes in spinal imaging during follow-up. However, a recent systematic review of 8 trials suggested that clinician educational interventions are unlikely to be associated with improved guideline-concordant imaging for low back pain.^13^ Our results support this conclusion. Systems-level interventions, such as point-of-care decision support^33^ or reimbursement restrictions for low-value imaging,^34^ may be more promising.

It is difficult to judge from the trial data whether the intervention positively affected clinician communication. During announced SPI visits during follow-up, intervention clinicians had significantly higher ratings on eliciting the patients’ perspective and conveying empathy. On the other hand, we found no significant difference in patient experience ratings among actual patients seen by intervention vs control physicians or in a post hoc analysis among patient respondents after visits for acute low back pain. While the large effect of the intervention on conveying empathy during SPI visits is impressive, the visits were announced and unblinded; thus, this finding should be interpreted cautiously.

We assessed as a secondary outcome the possibility that intervention effects might have generalized to imaging outcomes in patients with acute neck pain. Among these patients, the intervention was not associated with a significant difference in overall cervical spinal imaging during follow-up, although patients seeing intervention clinicians had significantly higher rates of cervical MRI or CT compared with those seeing control clinicians. While this association could represent an unanticipated intervention effect, analyses of secondary outcomes did not correct for multiplicity, warranting cautious interpretation.

### Limitations

This study has limitations. The rates of lumbar spinal imaging during the preintervention period among study clinicians were lower than anticipated and in national samples,^10,11^ which may have reduced study power. Both intervention and control clinicians recommended conservative measures and a watchful waiting approach in most of the announced SPI visits, and both ceiling effects (in these measures) and floor effects (in lumbar spinal imaging rates) may have been operative. It is conceivable that the intervention may have a more powerful effect on imaging among clinicians with higher baseline rates of imaging. We randomized at the clinician level rather than clinic level, and intervention clinicians may have communicated intervention content to control clinicians, leading to some degree of contamination. However, the lack of a significant preintervention vs postintervention change in the primary outcome of lumbar imaging within the intervention group suggests a null effect. The study was conducted during the COVID-19 pandemic, which may have affected patient-clinician communication and imaging utilization in the intervention and control arms. Our clinician sample also was derived from 2 health systems in the Sacramento area serving predominately insured populations, and results may not generalize to other settings. We adjusted analyses for some postrandomization imbalances in physician characteristics, but unmeasured confounding by physician or patient characteristics was possible.

## Conclusions

In this randomized clinical trial of an SPI-based educational intervention emphasizing clinician communication, the intervention had no effect on the primary outcome of spinal imaging among patients with low back pain. Although the intervention was rated highly by clinicians and was associated with more empathic communication during an announced follow-up standardized patient visit, the findings indicate that educational interventions emphasizing clinician communication are unlikely to reduce rates of low-value spinal imaging among primary care clinicians.

## Notes

Supplement 1.
Trial Protocol

Supplement 2.
eMethods.
**eTable 1.** Intervention Model With Key Skills and Criteria for Fulfillment **eFigure.** Figure Shared With Clinicians During Intervention Visits **eTable 2.** *ICD-10-CM* Codes for Lower Back Pain **eTable 3.** Neck Pain Diagnosis Codes **eTable 4.** Other Exclusions for Back or Neck Pain Imaging Measure **eTable 5.** Clinician Satisfaction With Standardized Patient Instructor Training **eTable 6.** Posttrial Attitudes and Use of Watchful Waiting by Study Arm
eReferences

Supplement 3.
Data Sharing Statement
