# Supplementary material for: Standardized Patient Communication and Low-Value Spinal Imaging: A Randomized Clinical Trial
Source: JAMA Netw Open. 2024 Nov 6;7(11):e2441826. doi: 10.1001/jamanetworkopen.2024.41826 (PMC11541634; doi:10.1001/jamanetworkopen.2024.41826)
Supplement: Supplement 1. — Trial Protocol [file jamanetwopen-e2441826-s001.pdf]

1) Protocol Title

Title: **Watchful Waiting as a Strategy for Reducing Low-value Spinal Imaging**

Protocol Version Date: *April 4, 2023*

2) Objectives

Spinal imaging in patients with acute low back pain poses risks from diagnostic evaluation of false-positive findings, patient labeling and anxiety, and unnecessary treatment (including spinal surgery) with potential downstream complications. Watchful waiting advice has been found an effective strategy to reduce low-value *treatment* (e.g., pediatric ear infections), and some evidence suggests a watchful waiting approach would be acceptable to many patients requesting tests. Meanwhile, psychological theory suggests that clinician messages could be tailored to magnify patient acceptance of a watchful waiting strategy.

We propose to refine and evaluate a novel simulated intervention using standardized patients (SPs) -- or actors playing the roles of patients – to teach clinicians to endorse a watchful waiting approach when patients request low-value spinal imaging for low back pain. Specific aims are:

Aim 1: **To use key informant interviews of front-line clinicians and focus groups with patients to refine a theory-informed standardized patient-based intervention designed to teach practicing clinicians how to advise watchful waiting when patients request low-value spinal imaging for low back pain.**

Aim 2: **To test the effectiveness of standardized patient instructor (SPI)-delivered clinician training in the use of watchful waiting in a randomized clinical trial.**

We hypothesize that the intervention will: a) reduce rates of low-value spinal imaging among actual patients with acute back pain seen by clinicians post-intervention (adjusting for pre-RCT rates); b) increase clinician advice to pursue watchful waiting during a followup visit with a SP; c) increase clinician self-reported use and efficacy of advising watchful waiting with actual low back pain patients; and d) have no adverse impact on actual patient trust and satisfaction with clinicians.

Aim 3: **To assess whether the intervention effects generalize to other high-cost, low-value imaging tests.**

We hypothesize that the SP intervention will: a) decrease rates of advanced neck imaging among actual patients with neck pain seen by study clinicians during the follow-up period (adjusting for baseline rates), and b) increase clininian self-reported use and efficacy of advising watchful waiting rather than imaging for patients with neck pain and other musculoskeletal pain.

3) Background

Early spinal imaging to evaluate acute back pain has become an accepted indicator of low-value care. Early spinal imaging provides no benefit

but poses potential patient safety risks deriving from the evaluation of false-positive tests and may pose the risk of unnecessary spinal surgery.

Primary care-based interventions to encourage a "watchful waiting" approach have shown promise in the setting of acute urinary infections, prostate cancer treatment, and in the diagnosis and evaluation of non-specific symptoms such as fatigue. We have collected promising preliminary data that a watchful waiting message is strongly associated with reduced likelihood of ordering low-value spinal imaging in primary care visits with standardized patients with low back pain. The current study would test a standardized patient-based intervention designed specifically to promote clinician use of a watchful waiting message for patients with acute or subacute low back pain.

4) Inclusion and Exclusion Criteria

To accomplish our specific aims, we use the following inclusion and exclusion criteria to recruit or to sample subjects for the following study phases:

A) Pre-trial key informant interviews focus groups

We will conduct clinician key informant interviews and patient focus groups to assist in intervention development prior to the randomized clinical trial (RCT).

For clinicians, we will recruit 10-12 clinicians who are in active primary or urgent care practice, serving adult patients. The principal investigator will identify eligible clinicians and invite them to participate by word or mouth or email. We will recruit clinicians both inside and outside the UCD system. We will ask the clinicians to participate in a 30-60 minute phone discussion about the preliminary intervention to assist us in its design.

To achieve data saturation with the patient focus groups, we will recruit up to 72 patients aged 18 to 65 years who have seen a clinician for back pain in the past 2 years. We anticipate the number of participants per focus group will vary due to attrition, but aim to enroll 6-12 participants per group. Recruitment flyers may be posted in local clinics or other public spaces as well as Craigslist online. Recruited patients will be asked to participate in a focus group to discuss the general issue of imaging for low back pain and specifically potential patient reactions to watchful waiting messages, as described in the patient focus group telephone screener script. We will not recruit patients over the age 65 years, because the prevalence of higher-risk back pain conditions rises with age, for which early spinal imaging may often be appropriate. Likewise, we will exclude patients with a history of spinal surgery or chronic, persistent back pain as imaging is generally appropriate. Because we want to elicit patient opinions about omitting imaging when it is low-value, we expect it will most useful to elicit opinions from younger patients with acute or subacute back pain.

In addition to age greater than 65 years, patients will be excluded if they cannot speak and understand English or lack the cognitive ability to engage in a focus group.

B) Randomized trial of the standardized patient (SP) intervention

Using our findings from the pre-trial clinician key informant interviews and patient focus groups, we have developed a standardized patient (SP) intervention designed to improve clinician skill and confidence in implementing a watchful waiting approach to spinal imaging among patients with acute low back pain. To test the effectiveness of the SP intervention, we will recruit 55 clinicians in active practice within the UC Davis or Sutter health systems. Clinicians will be eligible if they intend to practice either full-time or at least 50% full-time equivalent (FTE) in adult primary or urgent care in one of the target clinics for the trial duration and have been in active full-time or 50% FTE practice within the health system for at least two years prior to enrollment (to enable collection of baseline testing rates). We will not include clinicians practicing <50% FTE because of the need to accrue sufficient numbers of actual back pain patients during pre- and post-intervention phases for well-powered analyses of the primary outcome of any spinal imaging within 28 days among patients seen (in-person or remotely, e.g. video visit) with acute low back pain by study clinicians. We will exclude clinicians if they participated in pre-RCT key informant interviews. To achieve the desired sample size, we anticipate recruiting clinicians from up to 10 total primary care or urgent care clinics in the two systems.

C) Inclusion criteria for outcome assessment among actual patients of study clinicians

Primary outcomes will be spinal imaging ordering and completion rates among actual acute low back pain patients seen by study clinicians (in-person or remotely (e.g. video visits)) during the 18 month follow-up period after the study intervention. Secondary outcomes will include neck imaging ordering and completion among patients with neck pain diagnoses, and among all adult patients seen during the study period, the ordering and completion of any diagnostic test. Diagnostic tests for this latter outcome will consist of any test on a list of the most commonly ordered 498 diagnostic by UCD primary care clinicians, as we have identified using SlicerDicer. We will use electronic medical record (EMR) data to identify patients seen by study clinicians during this period as well as during a 2-year baseline period (to allow adjustment for baseline rates). We will submit a request to Bioinformatics for EMR data retrieval services for patients who meet the following criteria: age 18-90 years on the visit date, having a visit (in-person or video visit) with a study clinician, and an ICD-9/10 diagnosis for back pain with not similar diagnosis within the past 6 months (ICD-9-CM: 723.1, 724.x, or ICD-10: M54.2, M54.5, M54.6, M54.89), consistent with the HEDIS overuse measure related to low back pain imaging. Patients will be considered to have had spinal imaging if they completed plain spinal x-rays or lumbar MRI or CT within 28 days of their initial low back pain encounter. We will similarly request information on: 1) patients seen in-person or remotely by each clinician for acute neck pain to assess post-intervention rates of any diagnostic imaging (including laboratory, imaging, or other diagnostic tests). We will measure neck imaging and any diagnostic testing to examine the potential for the intervention effects to generalize beyond the management of low back pain (Aim 3).

5) Study Timelines

Our overall study has a four-year timeline, but participants in each of the phases above will have the following durations of partipation.

1) Clinicians and patients in interviews and focus groups will participate only for the duration of the interview or focus group.

2) Clinicians enrolled in the RCT will participate for approximately one year from the date of consent, allowing time for SP training and evaluation visits and a six-month post-RCT survey.

3) Patients with back or neck pain identified retrospectively using EMR will not actively participate in the research.

We estimate that study data will be collected within 2.5 years of project beginning and that the primary analyses will be completed by 3.5 years after study commencement.

6) Study Endpoints

We will assess study outcomes using four data sources: 1) electronic medical records of actual patients managed in-person or remotely by study clinicians; 2) audio-recordings of SP visits during the followup period; 3) a post-RCT survey of study clinicians; and 4) patient experience data collected from actual patinets of study clinicians by each healthcare institution.

Spinal imaging among actual clinician patients seen in-person or remotely via video visit: The primary outcome will be the rate of spinal imaging (xray, CT, or MRI) ordered and completed among patients aged 18-65 years with acute back pain seen by study clinicians during the 18 month followup period adjusted for the baseline imaging rate during the 24 month pre-randomization phase. To identify this outcome, we will collect automated data from the electronic medical records of all patients seen during an in-person office visit or via video visit by study clinicians during the 18 month post-intervention followup period, as well as the two year period prior to the intervention (to allow for adjustment for baseline utilization). We will also collect longitudinal data on ICD-9/10 diagnoses to allow identification of subsets of patients presenting with acute back pain based on the absence of back pain diagnoses on visits in the prior six months (ICD-9-CM: 723.1, 724.x, or ICD-10: M54.2, M54.5, M54.6, M54.89), consistent with the HEDIS overuse measure related to low back pain imaging. We will also collect patient-level covariates to enable stratified analyses and adjustment (e.g. age, sex, available race/ethnicity, any Medicaid insurance). We will also assess post-intervention rates of clinician ordering of both plain film and advanced neck imaging (MRI/CT) for patients seen in-person or remotely. The primary and secondary study endpoints will be assessed by abstracting automated visit data received from the study sites' EMR data analysts. An additional secondary outcome will be the rate of any diagnostic testing during the follow-up period among adult patients aged 18 years and older, including the 498 most commonly ordered tests by primary care clinicians, adjusted for baseline rates. Both UC Davis and Sutter utilize the same EMR system (Epic 2016), and so we expect to be able to harmonize patient and visit EMR data extracted from the two systems.

Audio-recordings of SP visits: We will interpret clinician implementation of watchful waiting advice during SP visits as a *secondary outcome.* For enrolled clinicians, we will audiorecord a single post-intervention SP encounter within 3-6 months of final SPI visit among intervention clinicians.Intervention standardized patient instructor (SPI) visits will also be audiorecorded to monitor SPI fidelity to the intervention, but SPI visits will not be used to assess outcomes.

For follow-up SP visits, we will use iterative systematic content analysis of transcripts from audio-recorded visits to quantify how frequently clinicians engaged in the specific steps emphasized in the watchful waiting intervention, as well as the overall extent to which clinicians implemented watchful waiting. To guide coding, Drs. Gosdin and Fenton will develop a manual to guide trained research assistants in coding transcriptions while simultaneously listening to audio-recorded visits. Coders will be blinded to allocation of clinicians to intervention and control. Inter-observer agreement for coding targeted behaviors will be assessed using Cohen's kappa, and disagreements will be resolved by consensus or by review of the audio-recording by a third party. We have successfully used this process to assess clinician-patient interaction in other studies. Ultimately, this process will likely generate a summary scale expressing the extent to which clinicians engaged in watchful waiting during SP visits.

iii) Post-RCT clinician surveys: Six months after final SPI visits, we will survey randomized clinicians regarding the use of watchful waiting when actual back pain patients requested low-value spinal imaging. We will also survey clinicians regarding the use of watchful waiting for neck pain, other regional musculoskeletal pain syndromes (e.g., shoulder and knee pain), and in other contexts (e.g., when patients request antibiotics for sinusitis). For intervention clinicians, the survey will also inquire regarding clinicians views on the quality, acceptability, utility of the SPI training and their openness to receiving similar SPI training in the future to address challenges in patient-doctor communication. Clinician self-reported use of watchful waiting during the follow-up period will constitute a *secondary outcome* of the RCT.

iv) Intervention effects on patient experience: To address concerns that a watchful waiting strategy might undermine patient trust and confidence in clinicians, we will assess for potential adverse impacts of the intervention on patient experience. We will specifically link study clinicians to pre- and post-intervention patient experience data collected by the health systems as part of routine care. We have confirmed the feasibility of such linkage with UC Davis executives and the Sutter system. At UCD, these measures include visit-level Consumer Assessment of Healthcare Providers and Systems (CAHPS) or Press-Ganey survey results, which we will use to develop pre- and post-intervention summary measures of patient experience. At Sutter, patient satisfaction ratings are based on responses from surveys gathered by an independent surveyor, NRC Health, shortly after a person's doctor visit. Analyses will assess for adjusted differences in post-intervention patient experience measures among intervention clinicians as compared to control clinicians, after adjustment for baseline patient experience.

7) Procedures Involved

We will initially conduct a series of key informant interviews with front-line clinicians to understand the barriers and facilitators to implementing a watchful waiting approach to diagnostic imaging in patients with low back pain (Aim 1). The goal of these interviews is to ensure that the study intervention is feasible, acceptable, and relevant to patients. Simultaneously we will conduct

focus groups with patients with the goal of understanding how patients' may react to clinician recommendations to pursue a watchful waiting approach and specifically to elicit patient response to draft watchful waiting messages. Pre-RCT clinician key informant interview and patient focus group guides are attached; however, since qualitative research is an inherently inductive process, some of the questions are likely to be modified on the fly, and additional follow-up or probing questions are likely to arise, building on responses to previous questions. We will share focus group findings with clinicians and vice versa, in hopes of deepening insights, and will use emerging qualitative findings to to refine the intervention.

We will then test the intervention in a randomized trial among community clinicians, comparing rates of advanced imaging for low back pain among real patients and clinician use of watchful waiting during the post-intervention period (Aim 2). Clinicians will be randomized 1:1 to intervention and control groups.

Clinicians randomized to intervention will receive three visits over 3-6 months with a standardized patient instructor (SPI), an actor portraying a patient who is trained tol instruct clinicians in how to deliver a compelling watchful waiting message that satisfies patients' needs for reassurance and information (see attached SPI script). To remain flexible during the COVID-19 pandemic, all SP and SPI visits will be made available in two modalities (in-person or remote video visit) and will be conducted in accordance with current guidelines. All SPIs will be trained to portray a patient presenting with acute back pain, describe their symptoms, know and deny any "red flags," and will make a request for imaging (see attached role descriptions). Each SPI visit will focus on a particular step (or steps) in the watchful waiting approach (see attached figure). Both intervention and control clinicians will receive an evaluative SP visit during which the SP will portray a patient with acute back pain but without delivering any instruction; this visit will be used to assess for intervention effects on clinician communication behaviors and recommendations. Imaging rates will be computed for each clinician during the follow-up period (with adjustment for baseline rates). All clinicians will be asked to complete a baseline and post-RCT survey (both surveys are attached). Clinicians randomized to the control group will be offered, but not required to receive a truncated version of the training following the post-intervention period.

To assess for generalization of intervention effects (Aim 3), we will examine whether intervention is associated with reduced use of imaging for neck pain and overall diagnostic testing among real patients and clinician report of using watchful waiting for patients with conditions other than back pain as described in section 4C above.

8) Data and/or Specimen Management and Confidentiality

☒ I understand that the UC Davis Health Electronic Health Record (EMR/EPIC) also contains the clinical data for Marshall Medical Center (MMC). I understand that MMC patient data cannot be accessed for research purposes and that I must take the necessary steps to ensure that MMC data is not accessed, used, or disclosed for UC Davis Health research purposes.

a) If any identifiers will be stored, how long will they be kept?

Identifiers will be stored 5 years beyond the project period.

b) For data that is coded with a linking key, at what point will the linking key be destroyed?

A linking key will be destroyed 5 years beyond the project period, once all anlysis is complete and data are no longer being stored.

c) If this research is both federally funded and you are using identifiable data/specimens, please explain why this research cannot be completed using de-identified data/specimens:

The identifiers (i.e. dates) are needed for analysis. This will allow us to stratify the outcomes by varying interval (e.g. 0-6 months post intervention, 7-12, or 13-18 month post intervention). This is scientifically important, as if we detect any intervention effects, it will be essential to determine if intervention effects are durable and persist throughout the follow-up period.

A. Clinicians. We will obtain the following research material from each clinician: key informant interview information prior to the trial; a brief baseline survey of trial clinicians collecting demographic information and practice experience data; clinician use of watchful waiting during study SP visits, ascertained via coding of visit audio recordings; and clinician responses to post-RCT visit surveys. We will request from the patient experience department patient responses to six items derived from the individual visit version of the CAHPS Clinician & Group Survey or Press-Ganey outpatient surveys, as available. Four items derived from the CAHPS Physician Communication Composite and inquired respectively about whether the PCP: 1) gave easy to understand information; 2) knew important information about the patients' medical history; 3) showed respect for what the patient had to say; and 4) spent enough time with the patient. A fifth item inquired about whether the patient would recommend the PCP to family and friends, while the sixth item requested that the patient rate the doctor from 0 to 10 from worse to best possible doctor. The Press-Ganey and NRC Health surveys include similar questions related to the provider's level of concern for the patient's questions/worries, explanations for the problem/condition, effort to include the patient in decision-making, discussion about treatment, and the likelihoold of recommending the provider to others, We will provide the department a list of participating clinician names. The patient experience department will provide an encrypted, password protected file with patient-level responses to these or similar items for each participating clinician during the pre- and post-intervention periods. The dates of patient responses will also be included for proper assignment to study period (pre- or post-intervention). We will also request the following demographic data on the responding patients: age, sex, race/ethnicity, education, self-rated physical and mental health, and whether anyone assisted the patient in responding to the survey.

Risks associated with participation include:

(1) Disclosure of information. Because of our method for maintaining strict confidentiality, the risk is extremely low for clinician participants. Research materials will

be kept under lock and key in a secure location at the research coordinating site. Only a study ID will identify individual research material, and names of participants will be kept under lock and key in a separate room to assure confidentiality. All computerized information will be kept on password-protected, physically secured study computers.

(2) Adverse effects of participation. Clinicians may experience some stress or emotional discomfort when learning new interviewing approaches. Given that this is a "low stakes" intervention, and since study clinicians will be volunteers, we anticipate this effect will be minimal. It is also possible that clinician participation will result in a small adverse impact on clinical productivity and time efficiency on days when study SPs are inserted into the clinician schedules. Because the intervention will be tightly scripted and was developed to be delivered within the confines of usual outpatient visit lengths, these effects are again anticipated to be minimal. We have also arranged with the participating health systems to compensate clinicians for lost productivity by providing relative value unit (RVU) credits for research visits.

B. Patients. Patients who participate in focus groups will be asked to fill out a brief health/demographic questionnaire and answer general questions about their impressions of deferring imaging in the setting of acute back or neck pain. We will obtain a limited data set from EMR data on diagnostic testing among actual patients seen in-person or remotely by study clinicians in up two years prior to enrollment and two years after the SPI intervention. EMR data variables will include: visit date, age, sex, available race/ethnicity, any Medicaid insurance, whether imaging tests wered ordered and completed during visits with participating clinicians, and ICD-9-CM: 723.1, 724.x, or ICD-10-CM diagnostic codes: M54.2, M54.5, M54.6, M54.89. The EMR data will include visit dates and the clinician name to allow linkage to all other clinician data obtained/collected (e.g. patient experience data and clinician pre- and post-intervention surveys).

Risks associated with the study include:

(1) Disclosure of information. Questionnaires will not contain or ask for any identifiers. Focus group responses will be transcribed, and all patient identifying data (if disclosed during the focus group) will not be transcribed. All computerized information will be kept on password-protected, physically secured study computers. Any files linking study IDs to participant names will be saved separately from datasets and accessible only to appropriate study personnel. Datasets will be abstracted by Biomedical informatics analysts and will be transferred to the study team using encrypted file transfer protocols. The EMR data will be a limited data set that includes visit dates. Public reports of results will only include aggregate data and therefore, will not contain any identifiable information. Because we anticipate all cell sizes in public reports to be large, we do not anticipate any significant risks that results could inadvertendly be used to identify the health information of individual patients.

C. Participant consent or waivers thereof

We will obtain verbal informed consent from clinicians and patients who participate in the pre-RCT key informant interview or focus groups (see relevant HRP-502 consent forms).

We will obtain verbal informed consent from clinicians who participate in the RCT of the SP intervention. Due to the minimal risk nature of this study and uncertainty around COVID-19, we request a waiver of the requirement for signed consent,

We request a waiver of informed consent for patient EMR data as the data received from the Biomedical informatics analsyst will contain identifiers and would be impracticable to obtain consent.

D. Data analysis and statistical power

Analyses of the primary and secondary outcomes will be conducted using the intention-to-treat principle. Visits among actual patients seen in-person or remotely during the post-intervention period (nested within clinicians) will be the units of analyses. The primary outcome will be within-visit counts of imaging or diagnostic studies (i.e., plain lumbar x-rays, MRI or CT scans) ordered and completed among patients during the post-intervention period. We will similarly obtain baseline data for patients seen by randomized clinicians during a two-year pre-intervention period. Using a Generalized Linear Model (GLM) with a Poisson distribution and log link, we will test for intervention effects by testing for the significance of an interaction term between a categorical variable for intervention group and a covariate distinguishing pre- and post-intervention periods. Analyses will account for nesting of patients within clinicians and clinics.

We used the exemplary dataset method in SAS to assess the power for a difference-in-difference analysis for a binary outcome that is assumed to have a 25% incidence in the control condition and that the effect of the intervention would be to lower the incidence by 5 percentage points. On the basis of empirical analysis of related data, we assumed that the outcome would have residual within-clinic and within-clinic/within-doctor correlations of 1% and 4%, respectively. We assumed hypothesis testing would be 2-tailed, with the type-1 error controlled at 5%. With 8 clinics, 6 clinicians per clinic, and 92 patients per doctor (57 pre-intervention and 35 post-intervention, we would have 80.1% power to detect the effect of interest.

Study data will be retained for five years after the final publication that occurs at end of the four year project period. We anticipate that all study publications will be complete within five years of the end of the project period. So we anticipate preserving the study data until 2033, at which point we will destroy the study data. Only the study team will have access to the study data.

9) Provisions to Monitor the Data to Ensure the Safety of Subjects

Because this research poses minimal risk to subjects, we will not establish a data monitoring committee.

10) Withdrawal of Subjects

Participants will be allowed to withdraw their consent for participation at any time during the study. If a clinician withdraws from the trial, we will use data already collected from the clinician, unless he or she requests that we rescind data previously collected. The retention of as much study data as possible may allow an intention-to-treat analysis where the clinician data is analyzed as randomized.

If a clinician enrolls in the trial, completes one or more SP visits, but wishes to no longer have additional SP visits, we will seek clinician permission to retain the existing study data to allow an intention-to-treat analysis.

11) Risks to Subjects

As detailed in Section 8, this research poses minimal risk to subjects and consist primarily of small risks of breach of confidentiality stemming from disclosure of patient or participant information. We will undertake numerous safeguards to minimize this already minimal risk. Most important, we will protect the privacy of participants by safely guarding study data. Paper forms, such as consent forms, will be stored in locked cabinets within locked and monitored research offices. Electronic data and study documents will be stored on research office computers with protections and UC Davis firewall protections. EMR datasets will be developed by trained IT personnel and will be transmitted to research staff using encryptied file transfer protocols and then stored on protected, secured research computers.

12) Potential Benefits to Subjects

Pre-RCT subjects who participate in key informant interview or focus groups will derive no direct benefits from participation. They will however be compensated for their time with gift cards ($100 for clinicians and $30 for patients).

Clinicians who participate in the randomized trial may benefit by receiving instruction from SPIs in the watchful waiting techniques. This technique may increase clinician confidence in communication with patients who are requesting low-value diagnostic tests. Clinicians in the RCT will also be compensated in two ways: 1) each will receive $100 gift card on completion of the post-RCT survey (Sutter clinicians will receive stipends in the amount of $100 per their internal policies and guidelines); 2) each will also receive the equivalent of ~$125 per SP visit to compensate for lost productivity on account of research visits. The method of providing this compensation will be developed by Medical Directors at each of the two study sites but may involve crediting clinicians with relative value units (RVUs) as a means of ameliorating for missed clinical charges.

13) Multi-Site Research

This study will be conducted within the UC Davis Primary Care Network and Sutter Health network, both in the Sacramento region. The primary site is UC Davis, where the site PI is Dr. Joshua Fenton. The Sutter site PI is Dr. Andrew Hudnut. Drs. Fenton and Hudnut will meet regularly throughout the study to monitor research activities, assure adherence to IRB approved protocols, and to communicate results.

UC Davis will be the repository of all study data. Sutter research staff will collect some data items, including clinician baseline and post-RCT surveys and

patient imaging data. Completed survey forms will be hand-entered into RedCAP by research staff if the clinician does not complete the surveys online.

Consistent with AHRQ's Single IRB policy, the project will rely on the UC Davis IRB. All UC Davis and Sutter research staff will be up-to-date with CITI training in human subjects research.

We will conduct regular meetings by conference call with site PIs and site research coordinators. All project milestones including study closure will be discussed and agreed upon during these meetings.

14) Community-Based Participatory Research

NA

15) Sharing of Results with Subjects

The study results will not be shared with participants, although results will be published in publicly accessible journals.

16) Prior Approvals

The study will receive IRB approval from UC Davis.

17) Provisions to Protect the Privacy Interests of Subjects

Research staff will be trained to make an effort to respect patients' and clinicians' privacy concerns both during recruitment and throughout the conduct of the study.

During recruitment of clinicians (for key informant interviews and trial participation) and patients (for focus groups), study staff will clearly explain that participation is voluntary and will describe procedures designed to protect participant privacy (e.g., use of study identification numbers on study documents, that only study staff will have access to study documents, that transcripts of interviews will contain no identifiers). Staff will also assure potential participants that study reports will contain no identifying information.

While spinal imaging for low back pain is not an emotionally charged topic, some patients may experience intense emotions during focus groups. Our skilled, experienced focus group leaders will respond with sensitivity and tact and guide discussions in a manner that put participants at ease.

18) Compensation for Research-Related Injury

NA

19) Economic Burden to Subjects

None

20) Drugs or Devices

Not applicable to this study

☐ I confirm that all investigational drugs will be received by the Investigational Drug Service (IDS). The IDS will store, handle, and administer those drugs so that they will be used only on subjects and be used only by authorized investigators.

☐ I confirm that all investigational devices will be labelled in accordance with FDA regulations and stored and dispensed in such a manner that they will be used only on subjects and be used only by authorized investigators.

21) Review Requirements

Are there any contractual obligations or other considerations that require IRB review of this research, or review at intervals other than those required by the Common Rule or FDA? If yes, check box:

☐ Yes

X No
