# Supplementary material for: Standardized Patient Communication and Low-Value Spinal Imaging: A Randomized Clinical Trial
Source: JAMA Netw Open. 2024 Nov 6;7(11):e2441826. doi: 10.1001/jamanetworkopen.2024.41826 (PMC11541634; doi:10.1001/jamanetworkopen.2024.41826)
Supplement: Supplement 2. — eMethods. eTable 1. Intervention Model With Key Skills and Criteria for Fulfillment eFigure. Figure Shared With Clinicians During Intervention Visits eTable 2. ICD-10-CM Codes for Lower Back Pain eTable 3. Neck Pain Diagnosis Codes eTable 4. Other Exclusions for Back or Neck Pain Imaging Measure eTable 5. Clinician Satisfaction With Standardized Patient Instructor Training eTable 6. Posttrial Attitudes and Use of Watchful Waiting by Study Arm eReferences [file jamanetwopen-e2441826-s002.pdf]

Supplementary Online Content

Fenton JJ, Cipri C, Gosdin M, et al. Standardized patient communication and low-value spinal imaging: a randomized clinical trial. *JAMA Netw Open*. 2024;7(10):e2441826. doi:10.1001/jamanetworkopen.2024.41826

eMethods

eTable 1. Intervention Model With Key Skills and Criteria for Fulfillment

eFigure 1. Figure Shared With Clinicians During Intervention Visits

eTable 2. *ICD-10-CM* Codes for Lower Back Pain

eTable 3. Neck Pain Diagnosis Codes

eTable 4. Other Exclusions for Back or Neck Pain Imaging Measure

eTable 5. Clinician Satisfaction With Standardized Patient Instructor Training

eTable 6. Posttrial Attitudes and Use of Watchful Waiting by Study Arm

eReferences

This supplementary material has been provided by the authors to give readers additional information about their work.

© 2024 Fenton JJ et al. *JAMA Network Open*.

eMethods

Outcome Ascertainment using Electronic Medical Record Data

In each of the two health systems, we used EpicCare EMR data to ascertain primary and secondary imaging outcomes among actual patients of study clinicians. We assessed imaging outcomes during a post-intervention period of up to 18 months after the final intervention SPI visit (or a randomly selected "pseudo-intervention" date among control clinicians). We also assessed imaging outcomes during a 24-month pre-intervention period (prior to the date of randomization) to enable adjustment for clinicians' baseline propensity to order imaging and to allow assessment for pre-to-post-intervention changes in imaging rates. Depending on the date of clinician randomization, the pre-intervention period ranged from March 22, 2019 to August 5, 2021, and the post-intervention period ranged from October 28, 2021 to May 31, 2023.

Patient visits included both in-person and telemedicine visits (with telemedicine visits identified using Healthcare Common Procedure Coding System codes G2010, G2012, G2061-2063; Current Procedural Terminology codes 99421-99423, 99431, 99441-99443; 99201-99215 with a GT-95 modifier). Consistent with the National Committee for Quality Assurance overuse measure related to low back pain imaging,[1] we used International Classification of Diseases (ICD)-10-Clinical Modification (CM) diagnosis codes to identify patients aged 18-90 years presenting with acute or new-onset back pain based on the absence of back pain diagnoses on visits in the prior six months and the inclusion of a back pain diagnostic code in the primary position on the claims (eTable 2). We also collected patient age and sex to allow adjustment for patient characteristics and restriction of analyses to patients aged 18-65 years consistent with the NCQA measure. Analogously, we identified pre- and post-intervention visits for acute neck pain based on ICD-10-CM codes for neck pain (eTable 3). Consistent with the NCQA measure,[1] we excluded patients with high-risk conditions that would justify spinal imaging by using diagnostic, procedural, and filled prescription data to identify and exclude patients with any of the following diagnoses or conditions during the study period: non-skin cancer or malignancy, human immunodeficiency virus infection, kidney or major organ transplant, osteoporosis therapy or dispensed prescription, fragility fracture, spinal infection, or 90 or more cumulative days of glucocorticoid prescriptions dispensed (eTable 4). Patients with visits who subsequently died during the study period were excluded from all imaging analyses.

Among eligible low back pain visits, we ascertained whether spinal imaging was ordered during the visit and whether it was completed within 90 days. We also assessed completion of advanced spinal imaging using Magnetic Resonance Imaging (MRI) or computed tomography (CT) within 90 days of eligible low back pain visits. We identified analogous cervical spinal imaging outcomes for patients with eligible acute neck pain visits.

Per protocol (NCT 04255199), we sought to measure the rate of overall diagnostic testing among adult patients adjusted for baseline by identifying counts of diagnostic tests ordered during primary care visits with participating clinicians during the 24-month period prior to their first SPI visits and from the date of their final SPI visits (or the "pseudo-intervention" date for control clinicians) through up to 18 months of follow-up. To facilitate this assessment, we identified the most common 500 diagnostic tests ordered during adult office visits in 2022 by primary care clinicians in one of the systems, including imaging tests, hematology and chemistry, urine and body fluid analyses, microbiology, electrocardiography, cardiac and nuclear medicine tests, but excluding tests performed for screening or prevention (e.g., lipid or diabetes mellitus tests, bilateral screening mammograms). However, due to differences in data storage at the two sites, we were not ultimately able to harmonize extractions of non-imaging tests. We therefore report only diagnostic imaging tests completed within 90 days of visits by adult patients aged 18-90 years study clinicians during the study period.

Summarizing Patient Experience Data at the Two Sites

Both health systems collected routine patient experience survey data after patient visits. However, the systems contracted with different survey vendors, and the vendors used different but comparable survey items related to the care provider.

At the first health system, five survey items pertained to the care provider: the provider's discussion of any proposed treatment (options, risks, benefits, etc.); the providers' efforts to include (the patient) in decisions about care; the concern the provider showed for questions or worries; explanations the provider gave about problems or conditions; and the likelihood of recommending the provider to others. The health system dichotomized each item as "top-box" or not based on whether the patient rated the provider at the highest response category on the item, and then summarized the provider performance on the visit as an average of the item ratings after assigning 100 points for each iterm that was top-box and 0 points for each item that was not top-box.

At the second health system, three survey items pertained to the care provider: whether the provider explained things clearly; whether the provider listened carefully; and the overall provider rating (from 0-10). Response options to the first two items were "yes, definitely," "yes, mostly," "yes, somewhat," and "no." To mirror the approach of the first health system, we assigned analogous top-box ratings for the three items (with 9 or 10 considered top-box for provider rating) and created a summary scale ranging from 0-100.

Within each system, we performed item-test and item-rest correlations and found that individual items had consistently high correlations with the summary scale. Using data within each system separately, we performed factor analyses that showed that the scale items loaded onto a single underlying factor. Cronbach's alpha for the summary scales were high within both health systems (α=0.95 in the first health system and 0.79 in the second health system). Based on these results, we concluded that the summary scale at each site likely reflected the same underlying construct ("patient experience with the provider"), so opted to pool the summary measures from the two sites in the analysis.

During the study period, the two systems received 21,799 responses to patient experience surveys after visits with study clinicians, including 6,868 (31.5%) in the pre-intervention period, 14,931 in the post-intervention period (68.5%), and 267 (1.2%) after visits with acute low back pain.

© 2024 Fenton JJ et al. *JAMA Network Open*.

eTable 1. Intervention Model with Key Skills and Criteria for Fulfillment

Step	Key Skills	Criteria for fulfillment with examples (to guide intervention content and coding)
1. Set the stage for deferred imaging by building trust	1. Demonstrate openness and interest<br>2. Avoid interruptions<br>3. Identify the patient's motivating concern or expectations	1. Non-verbal openness and engagement<br>• Sits, orients toward the patient<br>• Maintains open body position, leans in<br>• Frequent, attentive eye contact<br>• Engaged facial expressions or gestures (e.g., nodding)<br>2. Clinician doesn't interrupt early on. Allows patient to "tell their story" without cutting them off.<br>3. Clinician probes or asks for more information when patient signals a major underlying or motivating concern or expectations: "It sounds like you're worried that you seriously injured your back. Is that right?" or "You seem to be concerned that you need an MRI. Can you tell me more about that?"
2. Convey empathy	1. Legitimize patient's concerns<br>2. Name and explore patient's emotions<br>3. Express your understanding<br>4. Make supportive statements<br>5. Praise patient's attempts to address pain	1. Legitimizing statements: "I can understand why you're concerned."<br>2. Naming and exploring emotions: "You said you're afraid. Can you tell me more about what you're afraid of?"<br>3. Expressing understanding: "This obviously a tough thing to go through. I can see that it's really impacted your work life."<br>4. Supportive statements: "I'm committed to helping you find a workable solution."<br>5. Praise: "I think it's great that you've been trying to get out and walk."
3. Communicate optimism and openness while advocating a plan without imaging	1. Convey *optimism* when sharing your assessment and suggested plan, emphasizing reassuring aspects of the history and physical examination and the patient's favorable prognosis.<br>2. Advocate a conservative treatment plan without imaging<br>3. If patient asks about imaging, recommend a watchful waiting approach<br>4. Communicate your *availability* if the patient's pain doesn't improve.	1. Frames diagnosis and treatment recommendation in an optimistic, positive frame: "Overall, I'm actually quite reassured by your history and physical. I don't see any signs of a disc problem or nerve involvement, and I'm confident that your back pain is very likely to improve markedly over the next couple of weeks."<br>2. Confidently endorses an initial treatment plan that does not include imaging<br>EX: "Given your reassuring history and exam, I'm confident that you'll improve with conservative treatment, and in these cases, I don't recommend imaging at this time."<br>3. If patient asks about imaging, clinician advocates a "watchful waiting" approach:<br>EX: "I don't recommend imaging at this point, but I'd consider it in a few weeks if your pain didn't improve substantially, as I expect it to."<br>4. Articulates a follow-up, contingency plan for what the patient should do if the pain or other symptoms worsen or do not improve. Plan should address *how* the patient should contact the clinician, *when* they should do so, and *what* the clinician is likely to do in response. (The follow-up plan may or may not include a plan for deferred imaging.)<br>EX: "If you're pain is not substantially improved within two weeks, I'd like you to contact me via MyChart. I can then order you an x-ray and then we can have a either a phone call or a video visit."

© 2024 Fenton JJ et al. *JAMA Network Open*.

eFigure 1. Figure Shared with Clinicians During Intervention Visits

(Front)

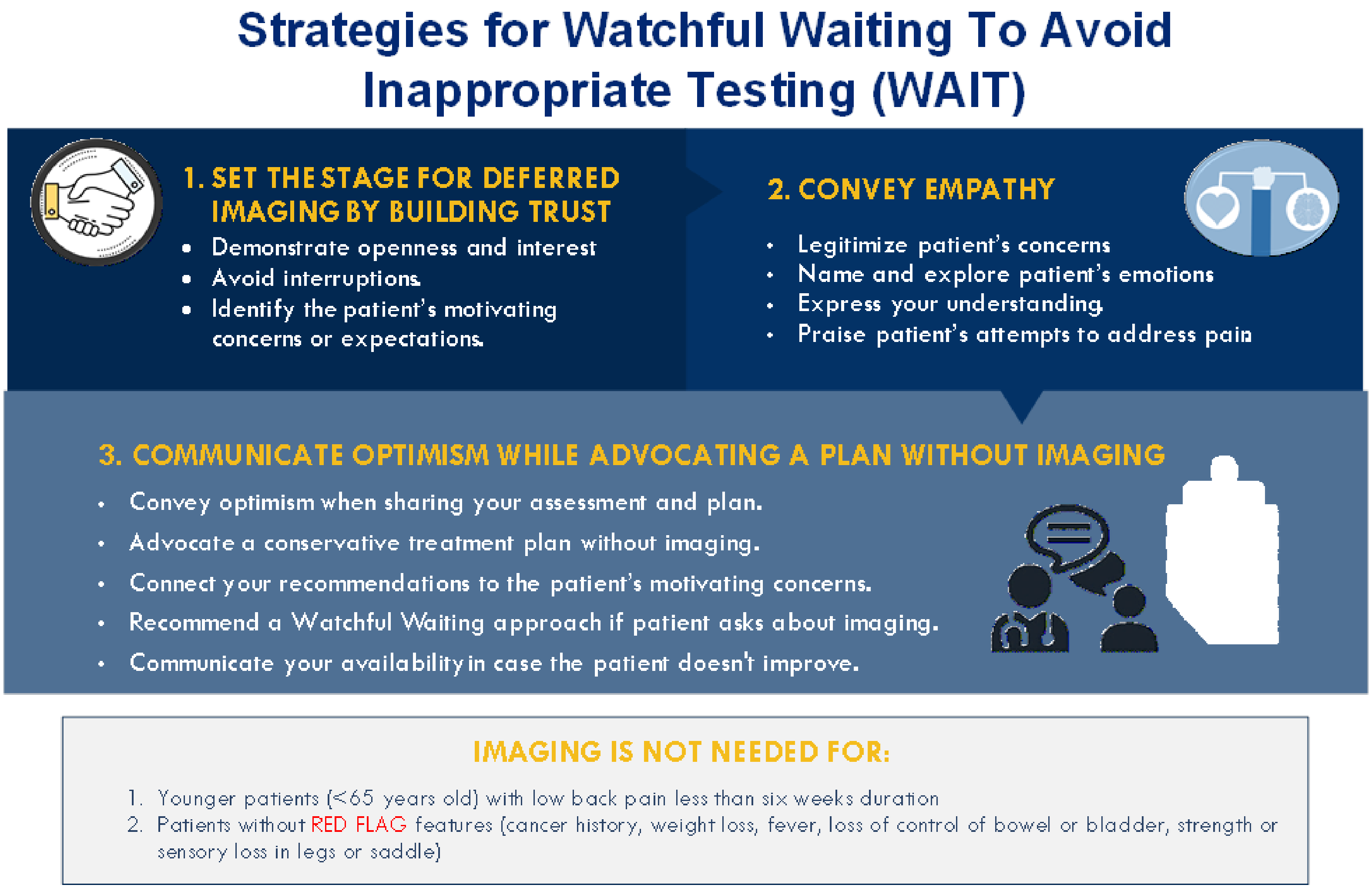

© 2024 Fenton JJ et al. *JAMA Network Open*.

(Back)

Example Communication Strategies

STEP 1

SET THE STAGE FOR DEFERRED IMAGING BY BUILDING TRUST

Ask about underlying concerns and expectations:

1. " You seem really worried about your pain. Tell me what worries you most."
2. "You seem to be concerned that you need an MRI. Can you tell me more about that?"

STEP 2

CONVEY EMPATHY

1. **Legitimizing statements:** "I can understand why you're concerned."
2. **Name and explore emotions:** "You said you're afraid. Can you tell me more about what you're afraid of?"
3. **Express understanding:** "This is a tough thing to go through. I can see it's really impacted your work life."
4. **Praise:** "I think it's great that you've been trying to get out and walk."

STEP 3

COMMUNICATE OPTIMISM WHILE ADVOCATING A PLAN WITHOUT IMAGING

1. **Optimistic framing of conservative treatment plan:** "Overall, I'm actually quite reassured by your history and physical. I don't see any signs of a disc problem or nerve involvement, and I'm confident that your back pain is very likely to improve markedly over the next couple of weeks with PT or perhaps a muscle relaxant."
2. **Connect treatment plan to patient's concerns**: "You expressed concerns about work. Gentle exercise and stretching will help maintain your back strength and flexibility so you can gradually get back to your normal daily activities and work."
3. **Employ Watchful Waiting:** "I don't recommend imaging at this point but I'd consider it in a few weeks if your pain didn't improve substantially, as I expect it to."
4. **Follow-up contingency plan:** "If your pain is not substantially improved within two weeks, I'd like you to contact me via MyChart. I can then order you an x-ray and we can have a either a phone call or a video visit."

© 2024 Fenton JJ et al. *JAMA Network Open*.

eTable 2. *ICD-10-CM* Codes for Lower Back Pain

ICD-10-CM	ICD-10-CM Description
M4305	Spondylolysis, thoracolumbar region
M4306	Spondylolysis, lumbar region
M4307	Spondylolysis, lumbosacral region
M4308	Spondylolysis, sacral and sacrococcygeal region
M4309	Spondylolysis, multiple sites in spine
M4315	Spondylolisthesis, thoracolumbar region
M4316	Spondylolisthesis, lumbar region
M4317	Spondylolisthesis, lumbosacral region
M4318	Spondylolisthesis, sacral and sacrococcygeal region
M4319	Spondylolisthesis, multiple sites in spine
M4605	Spinal enthesopathy, thoracolumbar region
M4606	Spinal enthesopathy, lumbar region
M4607	Spinal enthesopathy, lumbosacral region
M4608	Spinal enthesopathy, sacral and sacrococcygeal region
M4609	Spinal enthesopathy, multiple sites in spine
M4715	Other spondylosis with myelopathy, thoracolumbar region
M4716	Other spondylosis with myelopathy, lumbar region
M4725	Other spondylosis with radiculopathy, thoracolumbar region
M4726	Other spondylosis with radiculopathy, lumbar region
M4727	Other spondylosis with radiculopathy, lumbosacral region
M4728	Other spondylosis with radiculopathy, sacral and sacrococcygeal region
M47815	Spondylosis without myelopathy or radiculopathy, thoracolumbar region
M47816	Spondylosis without myelopathy or radiculopathy, lumbar region
M47817	Spondylosis without myelopathy or radiculopathy, lumbosacral region
M47818	Spondylosis without myelopathy or radiculopathy, sacral and sacrococcygeal region
M47895	Other spondylosis, thoracolumbar region
M47896	Other spondylosis, lumbar region
M47897	Other spondylosis, lumbosacral region
M47898	Other spondylosis, sacral and sacrococcygeal region
M4805	Spinal stenosis, thoracolumbar region
M48061	Spinal stenosis, lumbar region without neurogenic claudication
M48062	Spinal stenosis, lumbar region with neurogenic claudication
M4807	Spinal stenosis, lumbosacral region
M4808	Spinal stenosis, sacral and sacrococcygeal region
M488X5	Other specified spondylopathies, thoracolumbar region
M488X6	Other specified spondylopathies, lumbar region
M488X7	Other specified spondylopathies, lumbosacral region
M488X8	Other specified spondylopathies, sacral and sacrococcygeal region
M4985	Spondylopathy in diseases classified elsewhere, thoracolumbar region
M4986	Spondylopathy in diseases classified elsewhere, lumbar region
M4987	Spondylopathy in diseases classified elsewhere, lumbosacral region

© 2024 Fenton JJ et al. *JAMA Network Open*.

M4988	Spondylopathy in diseases classified elsewhere, sacral and sacrococcygeal region
M4989	Spondylopathy in diseases classified elsewhere, multiple sites in spine
M5105	Intervertebral disc disorders with myelopathy, thoracolumbar region
M5106	Intervertebral disc disorders with myelopathy, lumbar region
M5115	Intervertebral disc disorders with radiculopathy, thoracolumbar region
M5116	Intervertebral disc disorders with radiculopathy, lumbar region
M5117	Intervertebral disc disorders with radiculopathy, lumbosacral region
M5125	Other intervertebral disc displacement, thoracolumbar region
M5126	Other intervertebral disc displacement, lumbar region
M5127	Other intervertebral disc displacement, lumbosacral region
M5134	Other intervertebral disc degeneration, thoracic region
M5135	Other intervertebral disc degeneration, thoracolumbar region
M5136	Other intervertebral disc degeneration, lumbar region
M5137	Other intervertebral disc degeneration, lumbosacral region
M5145	Schmorl's nodes, thoracolumbar region
M5146	Schmorl's nodes, lumbar region
M5147	Schmorl's nodes, lumbosacral region
M5185	Other intervertebral disc disorders, thoracolumbar region
M5186	Other intervertebral disc disorders, lumbar region
M5187	Other intervertebral disc disorders, lumbosacral region
M519	Unspecified thoracic, thoracolumbar and lumbosacral intervertebral disc disorder
M533	Sacrococcygeal disorders, not elsewhere classified
M5385	Other specified dorsopathies, thoracolumbar region
M5386	Other specified dorsopathies, lumbar region
M5387	Other specified dorsopathies, lumbosacral region
M5388	Other specified dorsopathies, sacral and sacrococcygeal region
M5415	Radiculopathy, thoracolumbar region
M5416	Radiculopathy, lumbar region
M5417	Radiculopathy, lumbosacral region
M5418	Radiculopathy, sacral and sacrococcygeal region
M5430	Sciatica, unspecified side
M5431	Sciatica, right side
M5432	Sciatica, left side
M5440	Lumbago with sciatica, unspecified side
M5441	Lumbago with sciatica, right side
M5442	Lumbago with sciatica, left side
M545	Low back pain
M5489	Other dorsalgia
M9903	Segmental and somatic dysfunction of lumbar region
M9904	Segmental and somatic dysfunction of sacral region
M9905	Segmental and somatic dysfunction of pelvic region
M9913	Subluxation complex (vertebral) of lumbar region
M9914	Subluxation complex (vertebral) of sacral region
M9915	Subluxation complex (vertebral) of pelvic region

© 2024 Fenton JJ et al. *JAMA Network Open*.

M9923	Subluxation stenosis of neural canal of lumbar region
M9924	Subluxation stenosis of neural canal of sacral region
M9925	Subluxation stenosis of neural canal of pelvic region
M9932	Osseous stenosis of neural canal of thoracic region
M9933	Osseous stenosis of neural canal of lumbar region
M9934	Osseous stenosis of neural canal of sacral region
M9935	Osseous stenosis of neural canal of pelvic region
M9943	Connective tissue stenosis of neural canal of lumbar region
M9944	Connective tissue stenosis of neural canal of sacral region
M9945	Connective tissue stenosis of neural canal of pelvic region
M9952	Intervertebral disc stenosis of neural canal of thoracic region
M9953	Intervertebral disc stenosis of neural canal of lumbar region
M9954	Intervertebral disc stenosis of neural canal of sacral region
M9955	Intervertebral disc stenosis of neural canal of pelvic region
M9963	Osseous and subluxation stenosis of intervertebral foramina of lumbar region
M9964	Osseous and subluxation stenosis of intervertebral foramina of sacral region
M9965	Osseous and subluxation stenosis of intervertebral foramina of pelvic region
M9972	Connective tissue and disc stenosis of intervertebral foramina of thoracic region
M9973	Connective tissue and disc stenosis of intervertebral foramina of lumbar region
M9974	Connective tissue and disc stenosis of intervertebral foramina of sacral region
M9975	Connective tissue and disc stenosis of intervertebral foramina of pelvic region
M9983	Other biomechanical lesions of lumbar region
M9984	Other biomechanical lesions of sacral region
M9985	Other biomechanical lesions of pelvic region
S330XXA	Traumatic rupture of lumbar intervertebral disc, initial encounter
S33100A	Subluxation of unspecified lumbar vertebra, initial encounter
S33110A	Subluxation of L1/L2 lumbar vertebra, initial encounter
S33120A	Subluxation of L2/L3 lumbar vertebra, initial encounter
S33130A	Subluxation of L3/L4 lumbar vertebra, initial encounter
S33140A	Subluxation of L4/L5 lumbar vertebra, initial encounter
S335XXA	Sprain of ligaments of lumbar spine, initial encounter
S336XXA	Sprain of sacroiliac joint, initial encounter
S338XXA	Sprain of other parts of lumbar spine and pelvis, initial encounter
S339XXA	Sprain of unspecified parts of lumbar spine and pelvis, initial encounter
S3421XA	Injury of nerve root of lumbar spine, initial encounter
S3422XA	Injury of nerve root of sacral spine, initial encounter
S344XXA	Injury of lumbosacral plexus, initial encounter
S39002A	Unspecified injury of muscle, fascia and tendon of lower back, initial encounter
S39012A	Strain of muscle, fascia and tendon of lower back, initial encounter
S39092A	Other injury of muscle, fascia and tendon of lower back, initial encounter
S3982XA	Other specified injuries of lower back, initial encounter
S3992XA	Unspecified injury of lower back, initial encounter

© 2024 Fenton JJ et al. *JAMA Network Open*.

eTable 3. Neck Pain Diagnosis Codes

ICD-10-CM	ICD-10-CM Description
M4301	Spondylolysis, occipito-atlanto-axial region
M4302	Spondylolysis, cervical region
M4303	Spondylolysis, cervicothoracic region
M4311	Spondylolisthesis, occipito-atlanto-axial region
M4312	Spondylolisthesis, cervical region
M4313	Spondylolisthesis, cervicothoracic region
M436	Torticollis
M4601	Spinal enthesopathy, occipito-atlanto-axial region
M4602	Spinal enthesopathy, cervical region
M4603	Spinal enthesopathy, cervicothoracic region
M4711	Other spondylosis with myelopathy, occipito-atlanto-axial region
M4712	Other spondylosis with myelopathy, cervical region
M4713	Other spondylosis with myelopathy, cervicothoracic region
M4721	Other spondylosis with radiculopathy, occipito-atlanto-axial region
M4722	Other spondylosis with radiculopathy, cervical region
M4723	Other spondylosis with radiculopathy, cervicothoracic region
M47811	Spondylosis without myelopathy or radiculopathy, occipito-atlanto-axial region
M47812	Spondylosis w/o myelopathy or radiculopathy, cervical region
M47813	Spondylosis without myelopathy or radiculopathy, cervicothoracic region
M47891	Other spondylosis, occipito-atlanto-axial region
M47892	Other spondylosis, cervical region
M47893	Other spondylosis, cervicothoracic region
M4801	Spinal stenosis, occipito-atlanto-axial region
M4802	Spinal stenosis, cervical region
M4803	Spinal stenosis, cervicothoracic region
M488X1	Other specified spondylopathies, occipito-atlanto-axial region
M488X2	Other specified spondylopathies, cervical region
M488X3	Other specified spondylopathies, cervicothoracic region
M4981	Spondylopathy in diseases classified elsewhere, occipito-atlanto-axial region
M4982	Spondylopathy in diseases classified elsewhere, cervical region
M4983	Spondylopathy in diseases classified elsewhere, cervicothoracic region
M5000	Cervical disc disorder with myelopathy, unsp cervical region
M5001	Cervical disc disorder with myelopathy, high cervical region
M50020	Cervical disc disorder with myelopathy, mid-cervical region, unspecified level
M50021	Cervical disc disorder at C4-C5 level with myelopathy
M50022	Cervical disc disorder at C5-C6 level with myelopathy
M50023	Cervical disc disorder at C6-C7 level with myelopathy
M5003	Cervical disc disorder with myelopathy, cervicothoracic region
M5010	Cervical disc disorder with radiculopathy, unspecified cervical region
M5011	Cervical disc disorder with radiculopathy, high cervical region
M50120	Mid-cervical disc disorder, unspecified level

© 2024 Fenton JJ et al. *JAMA Network Open*.

M50121	Cervical disc disorder at C4-C5 level with radiculopathy
M50122	Cervical disc disorder at C5-C6 level with radiculopathy
M50123	Cervical disc disorder at C6-C7 level with radiculopathy
M5013	Cervical disc disorder with radiculopathy, cervicothoracic region
M5020	Other cervical disc displacement, unsp cervical region
M5021	Other cervical disc displacement, high cervical region
M50220	Other cervical disc displacement, mid-cervical region, unspecified level
M50221	Other cervical disc displacement at C4-C5 level
M50222	Other cervical disc displacement at C5-C6 level
M50223	Other cervical disc displacement at C6-C7 level
M5023	Other cervical disc displacement, cervicothoracic region
M5030	Other cervical disc degeneration, unsp cervical region
M5031	Other cervical disc degeneration, high cervical region
M50320	Other cervical disc degeneration, mid-cervical region, unspecified level
M50321	Other cervical disc degeneration at C4-C5 level
M50322	Other cervical disc degeneration at C5-C6 level
M50323	Other cervical disc degeneration at C6-C7 level
M5033	Other cervical disc degeneration, cervicothoracic region
M5080	Other cervical disc disorders, unspecified cervical region
M5081	Other cervical disc disorders, high cervical region
M50820	Other cervical disc disorders, mid-cervical region, unspecified level
M50821	Other cervical disc disorders at C4-C5 level
M50822	Other cervical disc disorders at C5-C6 level
M50823	Other cervical disc disorders at C6-C7 level
M5083	Other cervical disc disorders, cervicothoracic region
M5090	Cervical disc disorder, unsp, unspecified cervical region
M5091	Cervical disc disorder, unspecified, high cervical region
M50920	Unspecified cervical disc disorder, mid-cervical region, unspecified level
M50921	Unspecified cervical disc disorder at C4-C5 level
M50922	Unspecified cervical disc disorder at C5-C6 level
M50923	Unspecified cervical disc disorder at C6-C7 level
M5093	Cervical disc disorder, unspecified, cervicothoracic region
M530	Cervicocranial syndrome
M531	Cervicobrachial syndrome
M5381	Other specified dorsopathies, occipito-atlanto-axial region
M5382	Other specified dorsopathies, cervical region
M5383	Other specified dorsopathies, cervicothoracic region
M5411	Radiculopathy, occipito-atlanto-axial region
M5412	Radiculopathy, cervical region
M5413	Radiculopathy, cervicothoracic region
M542	Cervicalgia
M5481	Occipital neuralgia
M9900	Segmental and somatic dysfunction of head region
M9901	Segmental and somatic dysfunction of cervical region

© 2024 Fenton JJ et al. *JAMA Network Open*.

M9910	Subluxation complex (vertebral) of head region
M9911	Subluxation complex (vertebral) of cervical region
M9920	Subluxation stenosis of neural canal of head region
M9921	Subluxation stenosis of neural canal of cervical region
M9930	Osseous stenosis of neural canal of head region
M9931	Osseous stenosis of neural canal of cervical region
M9940	Connective tissue stenosis of neural canal of head region
M9941	Connective tissue stenosis of neural canal of cervical region
M9950	Intervertebral disc stenosis of neural canal of head region
M9951	Intervertebral disc stenosis of neural canal of cervical region
M9960	Osseous and subluxation stenosis of intervertebral foramina of head region
M9961	Osseous and subluxation stenosis of intervertebral foramina of cervical region
M9970	Connective tissue and disc stenosis of intervertebral foramina of head region
M9971	Connective tissue and disc stenosis of intervertebral foramina of cervical region
M9980	Other biomechanical lesions of head region
M9981	Other biomechanical lesions of cervical region
S130XXA	Traumatic rupture of cervical intervertebral disc, initial encounter
S13100A	Subluxation of unspecified cervical vertebrae, initial encounter
S13110A	Subluxation of C0/C1 cervical vertebrae, initial encounter
S13120A	Subluxation of C1/C2 cervical vertebrae, initial encounter
S13130A	Subluxation of C2/C3 cervical vertebrae, initial encounter
S13140A	Subluxation of C3/C4 cervical vertebrae, initial encounter
S13150A	Subluxation of C4/C5 cervical vertebrae, initial encounter
S13160A	Subluxation of C5/C6 cervical vertebrae, initial encounter
S13170A	Subluxation of C6/C7 cervical vertebrae, initial encounter
S13180A	Subluxation of C7/T1 cervical vertebrae, initial encounter
S134XXA	Sprain of ligaments of cervical spine, initial encounter
S138XXA	Sprain of joints and ligaments of other parts of neck, initial encounter
S139XXA	Sprain of joints and ligaments of unspecified parts of neck, initial encounter
S142XXA	Injury of nerve root of cervical spine, initial encounter
S143XXA	Injury of brachial plexus, initial encounter
S161XXA	Strain of muscle, fascia and tendon at neck level, initial encounter
S168XXA	Other specified injury of muscle, fascia and tendon at neck level, initial encounter
S169XXA	Unspecified injury of muscle, fascia and tendon at neck level, initial encounter
S1980XA	Other specified injuries of unspecified part of neck, initial encounter
S1989XA	Other specified injuries of other specified part of neck, initial encounter
S199XXA	Unspecified injury of neck, initial encounter

© 2024 Fenton JJ et al. *JAMA Network Open*.

eTable 4. Other Exclusions for Back or Neck Pain Imaging Measure

Diagnosis/Condition	Diagnostic or Other Codes (ICD-10-CM, HCPCS, etc.)
Non-skin cancer or malignancy	C00.0-C00.6, C00.8, C00.9, C01, C02.0-C02.4, C02.8, C02.9, C03.0, C03.1, C03.9, C04.0, C04.1, C04.8, C04.9, C05.0, C05.1, C05.2, C05.8, C05.9, C06.0, C06.1, C06.2, C06.80, C06.89, C06.9, C07, C08.0, C08.1, C08.9, C09.0, C09.1, C09.8, C09.9, C10.0- C10.4, C10.8, C10.9, C11.0-C11.3, C11.8, C11.9, C12, C13.0, C13.1, C13.2, C13.8, C13.9, C14.0, C14.2, C14.8, C15.3, C15.4, C15.5, C15.8, C15.9, C16.0-C16.6, C16.8, C16.9, C17.0-C17.3, C17.8, C17.9, C18.0-C18.9, C19, C20, C21.0; C44.00-C44.02; C92.60, C93.92, C93.Z0, C93.Z1, C93.Z2, C94.00, C94.01, C94.02, C94.20, C94.21, C94.22, C94.30, C94.31, C94.32, C94.40, C94.41, C94.42, C94.6, C94.80, C94.81, C94.82, C95.00, C95.01, C95.02, C95.10, C95.11, C95.12, C95.90, C95.91, C95.92 C96.0, C96.2, C96.20, C96.21, C96.22, C96.29, C96.4, C96.5, C96.6, C96.9, C96.A, C96.Z;<br><br>D00.00- D00.08, D00.1, D00.2, D01.0- D01.3, D01.40, D01.49, D01.5, D01.7, D01.9, D02.0, D02.1, D02.20-D02.22, D02.3, D02.4, D03.0, D03.10, D03.11, D03.111, D03.112, D03.121, D03.122, D03.20, D03.21, D03.22, D03.30, D03.39, D03.4, D03.51, D03.52, D03.59, D03.60, D03.61, D03.62, D03.70, D03.71, D03.72, D03.8, D03.9, D04.0, D04.10, D04.11, D04.111, D04.112, D04.12, D04.121, D04.122, D04.20, D04.21, D04.22, D04.30, D04.39, D04.4, D04.5, D04.60, D04.61, D04.62, D04.70, D04.71, D04.72, D04.8, D04.9, D05.00, D05.01, D05.02, D05.10, D05.11, D05.12, D05.80, D05.81, D05.82, D05.90, D05.91, D05.92, D06.0, D06.1, D06.7, D06.9, D07.0, D07.1, D07.2, D07.30, D07.39, D07.4, D07.5, D07.60, D07.61, D07.69, D09.0, D09.10, D09.19, D09.20, D09.21, D09.22, D09.3, D09.8, D09.9, D37.01, D37.02, D37.030, D37.031, D37.032, D37.039, D37.04, D37.05, D37.09, D37.1- D37.6, D37.8, D37.9, D38.0-D38.6, D39.0, D39.10, D39.11, D39.12, D39.2, D39.8, D39.9, D40.0, D40.10, D40.11, D40.12, D40.8, D40.9, D41.00, D41.01, D41.02, D41.10, D41.11, D41.12, D41.20, D41.21, D41.22, D41.3, D41.4, D41.8, D41.9, D42.0, D42.1, D42.9, D43.0-D43.4, D43.8, D43.9, D44.0, D44.10, D44.11, D44.12, D44.2-D44.7, D44.9, D45, D46.0, D46.1, D46.20, D46.21, D46.22, D46.4, D46.9, D46.A, D46.B, D46.C, D46.Z, D47.0, D47.01, D47.02, D47.09-D47.4, D47.9, D47.Z1, D47.Z2, D47.Z9, D48.0-D48.5, D48.60, D48.61, D48.62, D48.7, D48.9, D49.0, D49.1, D49.2, D49.3, D49.4, D49.5, D49.511, D49.512, D49.519, D49.59, D49.6, D49.7, D49.81, D49.89, D49.9<br><br>Z85.00, Z85.01, Z85.020
HIV	B20, Z21
Kidney / Major organ transplant	History of Kidney Transplant: ICD-10-CM: Z94.0<br>Kidney Transplant:<br>CPT: 50360, 50365, 50380,<br>▪ HCPCS: S2065<br>▪ ICD-10-PCS: 0TY00Z0, 0TY00Z1, 0TY00Z2, 0TY10Z0, 0TY10Z1, 0TY10Z2<br>o Organ Transplant Other Than Kidney:<br>▪ CPT : 32850, 32851, 32852, 32853, 32854, 32855, 32856
Osteoporosis therapy or a dispensed prescription to treat osteoporosis	▪ HCPCS:<br>▪ J0897 Injection, denosumab, 1 mg<br>▪ J1740 Injection, ibandronate sodium, 1 mg<br>▪ J3110 Injection, teriparatide, 10 mcg<br>▪ J3111 Injection, romosozumab-aqqg, 1 mg

© 2024 Fenton JJ et al. *JAMA Network Open*.

	▪ J3489 Injection, zoledronic acid, 1 mg<br><br>Any prescriptions for:<br><br>• Alendronate<br>• Alendronate-cholecalciferol<br>• Ibandronate<br>• Risedronate<br>• Zoledronic Acid<br>• Abaloparatide<br>• Denosumab<br>• Raloxifene<br>• Romosozumab<br>• Teriparatide
Fragility fracture	ICD-10-CM: M48.40XA, M48.40XD, M48.40XG, M48.40XS, M48.41XA, M48.41XD, M48.41XG, M48.41XS, M48.42XA, M48.42XD, M48.42XG, M48.42XS, M48.43XA, M48.43XD, M48.43XG, M48.43XS, M48.44XA, M48.44XD, M48.44XG, M48.44XS, M48.45XA, M48.45XD, M48.45XG, M48.45XS, M48.46XA, M48.46XD, M48.46XG, M48.46XS, M48.47XA, M48.47XD, M48.47XG, M48.47XS, M48.48XA, M48.48XD, M48.48XG, M48.48XS, M80.08XA, M80.08XD, M80.08XG, M80.08XK, M80.08XP, M80.08XS, M80.88XA, M80.88XD, M80.88XG, M80.88XK, M80.88XP, M80.88XS, M84.359A, M84.359D, M84.359G, M84.359K, M84.359P, M84.359S, M97.01XA, M97.01XD, M97.01XS, M97.02XA, M97.02XD, M97.02XS
Spinal infection	A17.81, G06.1, M46.25-M46.28, M46.35-M46.38, M46.46-M46.48
Prolonged corticosteroids	During the entire study period, exclude patients with 90 or more consecutive days' supplied with one of the following oral drugs:<br><br>• Hydrocortisone<br>• Cortisone<br>• Prednisone<br>• Prednisolone<br>• Methylprednisolone<br>• Triamcinolone<br>• Dexamethasone<br>• Betamethasone

© 2024 Fenton JJ et al. *JAMA Network Open*.

eTable 5. Clinician Satisfaction With Standardized Patient Instructor Training (n=22 intervention clinicians)*

Item	Mean rating (SD)†	Range
Overall quality of training	1.4 (0.6)	1-3
Easy to understand	1.2 (0.4)	1-2
Would recommend to other doctors	1.7 (0.7)	1-3
Watchful waiting training relevant to my practice	1.3 (0.5)	1-2
Training helped me provide better care to my patients	1.9 (0.9)	1-4
Training improved my communication with patients	1.7 (0.9)	1-4

*22 of 25 intervention clinicians responded to the post-trial survey (88%).

†Response range for each item was: 1=Strongly agree, 2=Agree, 3=Neither agree nor disagree, 4=Disagree, 5=Strongly disagree

© 2024 Fenton JJ et al. *JAMA Network Open*.

eTable 6. Posttrial Attitudes and Use of Watchful Waiting by Study Arm

Item	Intervention (n=22) Mean (SD)*	Control (n=27) Mean (SD)*	p-value[†]
Watchful waiting is my preferred approach for most patients with low back pain	1.4 (0.5)	1.5 (0.6)	0.49
I use watchful waiting more often now than before study	2.2 (1.1)	3.0 (0.9)	0.005
I feel confident using watchful waiting for patients with low back pain	1.3 (0.5)	1.6 (0.6)	0.045
I know how to address concerns when patients are reluctant about watchful waiting	1.5 (0.5)	1.8 (0.4)	0.019
I believe patients are less satisfied with a watchful waiting approach	3.1 (1.1)	2.9 (0.9)	0.41

*22 of 25 intervention clinicians (88%) and 27 of 28 control clinicians (96%) responded to the post-trial survey. Response range for each item was: 1=Strongly agree, 2=Agree, 3=Neither agree nor disagree, 4=Disagree, 5=Strongly disagree

[†] T-test comparing means from intervention and control groups.

© 2024 Fenton JJ et al. *JAMA Network Open*.

eReferences

1. Use of Imaging Studies for Low Back Pain. National Committee on Quality Assurance. Accessed March 16, 2024. https://www.ncqa.org/hedis/measures/use-of-imaging-studies-for-low-back-pain/

2. Gerrity MS, DeVellis RF, Earp JA. Physicians' reactions to uncertainty in patient care. A new measure and new insights. *Med Care*. 1990;28(8):724-736.

© 2024 Fenton JJ et al. *JAMA Network Open*.
