# Supplementary material for: Standardized Patient Communication and Low-Value Spinal Imaging: A Randomized Clinical Trial
Source: JAMA Netw Open. 2024 Nov 6;7(11):e2441826. doi: 10.1001/jamanetworkopen.2024.41826 (PMC11541634; doi:10.1001/jamanetworkopen.2024.41826)
Supplement: Supplement 3. — Data Sharing Statement [file jamanetwopen-e2441826-s003.pdf]

Data Sharing Statement

Fenton. Standardized Patient Communication and Low-Value Spinal Imaging. *JAMA Netw Open*. Published October 30, 2024. doi:10.1001/jamanetworkopen.2024.41826

Data
Additional Information: NCT 04255199, ClinicalTrials.gov
Data available: No
